# Comparative Evaluation of Microleakage in Class II Composite Restorations Using Two Light-Cure Pulp Capping Agents as Cavity Liners: An In Vitro Study

**DOI:** 10.7759/cureus.83404

**Published:** 2025-05-03

**Authors:** Shirin Kshirsagar, Indraja S Deshmukh, Deepali Samel, Ved M Talathi, Rishikesh Meshram, Priyanka Razdan, Seema Gupta

**Affiliations:** 1 Department of Conservative Dentistry and Endodontics, Yogita Dental College and Hospital, Khed, IND; 2 Department of Conservative Dentistry and Endodontics, Swargiya Dadasaheb Kalmegh Smruti Dental College and Hospital, Nagpur, IND; 3 Department of Pediatric and Preventive Dentistry, Yogita Dental College and Hospital, Khed, IND; 4 Department of Orthodontics, Kothiwal Dental College and Research Centre, Moradabad, IND

**Keywords:** cavity liners, composite resin, in vitro, light-cured, pulp capping

## Abstract

Introduction: Reducing microleakage is crucial for maintaining the long-term efficacy of composite restorations. The selection of the cavity liner may affect both the marginal seal and the structural integrity of the restoration. This study aimed to assess and compare microleakage in class II composite restorations using two distinct light-cured pulp capping agents as cavity liners, analyzed under a stereomicroscope.

Materials and methods: Class II cavities with standardized dimensions were prepared for 60 noncarious human mandibular premolars. All the specimens were randomly allocated to three distinct groups, each containing 20 samples. In group 1 (n = 20), a self-etch bonding agent (Scotchbond Universal, 3M, Minnesota, USA) was uniformly applied to the entire cavity using an applicator tip and subsequently light-cured for 20 seconds. The cavities were then restored using composite resin (Filtek Z350 XT, 3M, Minnesota, USA) and light-cured for 30 seconds. In group 2 (n = 20), an approximately 0.8-mm-thick layer of TheraCal LC (Bisco Inc., Illinois, USA) was applied to the axial wall as a cavity liner. Group 3 (n = 20) involved the application of an approximately 0.8-mm-thick layer of Bio-LCB (Essential Dental Systems, New Jersey, USA) on the axial wall as a cavity liner. All tooth surfaces, except the 2-mm-wide area surrounding the edges of each restoration, were sealed with two nail polish coats. This was followed by a 48-hour immersion in 2% methylene blue dye. The teeth were then rinsed and sectioned longitudinally in the mesiodistal direction, coincident with the center of the restoration, using a slow-speed water-cooled diamond disc. To assess microleakage, the two tooth parts exhibiting dye penetration were examined under a stereomicroscope at 20x magnification (RSMr-6 series; Radical Scientific Equipment Pvt. Ltd., Haryana, India). Intergroup comparisons were performed using one-way analysis of variance (ANOVA), followed by post-hoc analysis using Tukey’s test.

Results: Statistically significant differences were observed between the groups (p < 0.05). Group 1 exhibited the highest mean microleakage values (3.35 ± 0.59), group 2 showed a moderate reduction in microleakage with a mean value of 2.30 ± 1.17, and group 3 had the lowest mean microleakage of 1.15 ± 0.93. Post-hoc analysis revealed significant differences between the groups. Groups 2 and 3 showed better results than group 1 (p < 0.05).

Conclusion: It can be concluded that Bio-LCB, as a light-cure pulp capping agent, showed minimal microleakage when used as a cavity liner in class II composite restorations.

## Introduction

Composite resin restorations remain the preferred choice in restorative dentistry owing to their superior aesthetic qualities and excellent adhesive properties. However, one of the major challenges associated with their use is polymerization shrinkage, which can significantly affect the marginal integrity of restorations [1]. During the curing process, the resin matrix undergoes volumetric contraction, leading to internal stresses at the interface between the tooth and the restoration. These stresses can result in microleakage, which facilitates the entry of oral fluids, bacteria, and their by-products into the restoration margins, potentially causing clinical complications [2,3].

Extensive carious lesions are managed using protective cavity liners that shield the dental pulp from external stimuli and promote the formation of reparative dentin [4]. These agents can occlude dentinal tubules and protect the pulp from microbial invasions, thermomechanical stresses, therapeutic interventions, and irritants [2]. Pulp capping agents are increasingly being utilized beneath composite restorations as cavity liners to counteract the adverse effects of polymerization shrinkage [4]. These materials act as protective barriers for the pulp and improve bond strength and marginal sealing [5]. Among the available options, light-cured calcium silicate-based agents, such as TheraCal LC (Bisco Inc., Illinois, USA) and Bio-LCB (Essential Dental Systems, New Jersey, USA), have shown considerable promise because of their convenient handling and bioactivity [6-8]. These materials release calcium ions, which contribute to dentin remineralization and the formation of a hydroxyapatite layer, thereby enhancing sealing efficacy at the restoration interface [8].

When pulp capping agents are not used, the effects of polymerization shrinkage become more pronounced. Without this intermediary layer, the stresses generated during polymerization can extend to the dentinal tubules and pulp tissue, increasing the likelihood of postoperative sensitivity, pulp inflammation, and microcracks in the dentin [9]. Furthermore, the lack of a barrier compromises the marginal seal, leading to microleakage, secondary caries, and potential restoration failure [3]. Traditional pulp capping materials, such as calcium hydroxide-based agents, have been used extensively but are associated with limitations. These include inadequate compressive strength, poor durability under composite restorations, and inability to form strong bonds with dentin or composites [10,11].

In contrast, light-cured pulp capping agents such as TheraCal LC and Bio-LCB have notable advantages. These include superior mechanical properties such as enhanced compressive strength and durability, making them better suited for stress-bearing composite restorations [11,12]. Additionally, their bioactivity facilitates dentin remineralization and promotes the development of a robust hydroxyapatite layer that improves sealing ability and reduces microleakage [13]. Notwithstanding their theoretical benefits, the relative effectiveness of these materials in mitigating microleakage could significantly impact their functionality in clinical settings. The purpose of this investigation was to assess and compare the microleakage associated with class II composite restorations using these two light-curing pulp capping agents as cavity liners. The null hypothesis posited for this study was that there would be no significant differences in microleakage between the groups.

## Materials and methods

Study design and setting

The present in vitro study was conducted at the Department of Conservative Dentistry and Endodontics at Yogita Dental College, Khed, between January 2025 and March 2025. The study was approved by the Institutional Ethical Committee and adhered to the Checklist for Reporting In Vitro Studies (CRIS) guidelines [14] and followed the principles of the Declaration of Helsinki.

Sample size estimation

The sample size estimation for this study was calculated using G*Power software version 3.6.9 (Heinrich Heine University Düsseldorf, Düsseldorf, Germany) with a power of 80% and an alpha error of 5%. An effect size of 0.56, derived from a reference study [15], was used to analyze the mean difference in dye microleakage between the study groups. Based on these parameters, the estimated sample size was determined to be 60, corresponding to 20 samples in each study group.

Specimen preparation

Sixty intact human single-rooted mandibular premolars, indicated for orthodontic or periodontal extraction, were selected for this study. The teeth were cleaned of calculus, soft tissue, and debris with hand instrumentation and stored in saline for a maximum of one month after extraction. The saline solution was changed every two days. Teeth with caries, restorations, hypoplasia, or fractures were also excluded. Standardized mesio-occlusal class II cavity preparations were performed using a No. 245 bur (Mani Inc., Haryana, India) with a high-speed air-water-cooled handpiece (NSK, Nakanishi Inc., Tochigi, Japan). The cavity dimensions were standardized to 4 mm buccolingually, with a pulpal depth of 2 mm, and the gingival seat positioned 1 mm coronal to the cementoenamel junction (CEJ).

Group allocation

All specimens were randomly allocated to three distinct groups. In group 1 (n = 20), a self-etch bonding agent (Scotchbond Universal, 3M, Minnesota, USA) was uniformly applied to the entire cavity using an applicator tip and subsequently light-cured for 20 seconds. The cavities were then restored using composite resin (Filtek Z350 XT, 3M, Minnesota, USA) and light-cured for 30 seconds. In group 2 (n = 20), an approximately 0.8-mm-thick layer of TheraCal LC was applied to the axial wall, followed by the application of a self-etch bonding agent and restoration with composite resin, mirroring the procedure of group 1. Group 3 involved the application of an approximately 0.8-mm-thick layer of Bio-LCB on the axial wall, followed by the application of the self-etch bonding agent and restoration with composite resin, consistent with the methodology employed in group 1. A Tofflemire matrix band retainer was used during preparation to prevent gingival overhang of the restoration.

Methodology

**Figure 1:**
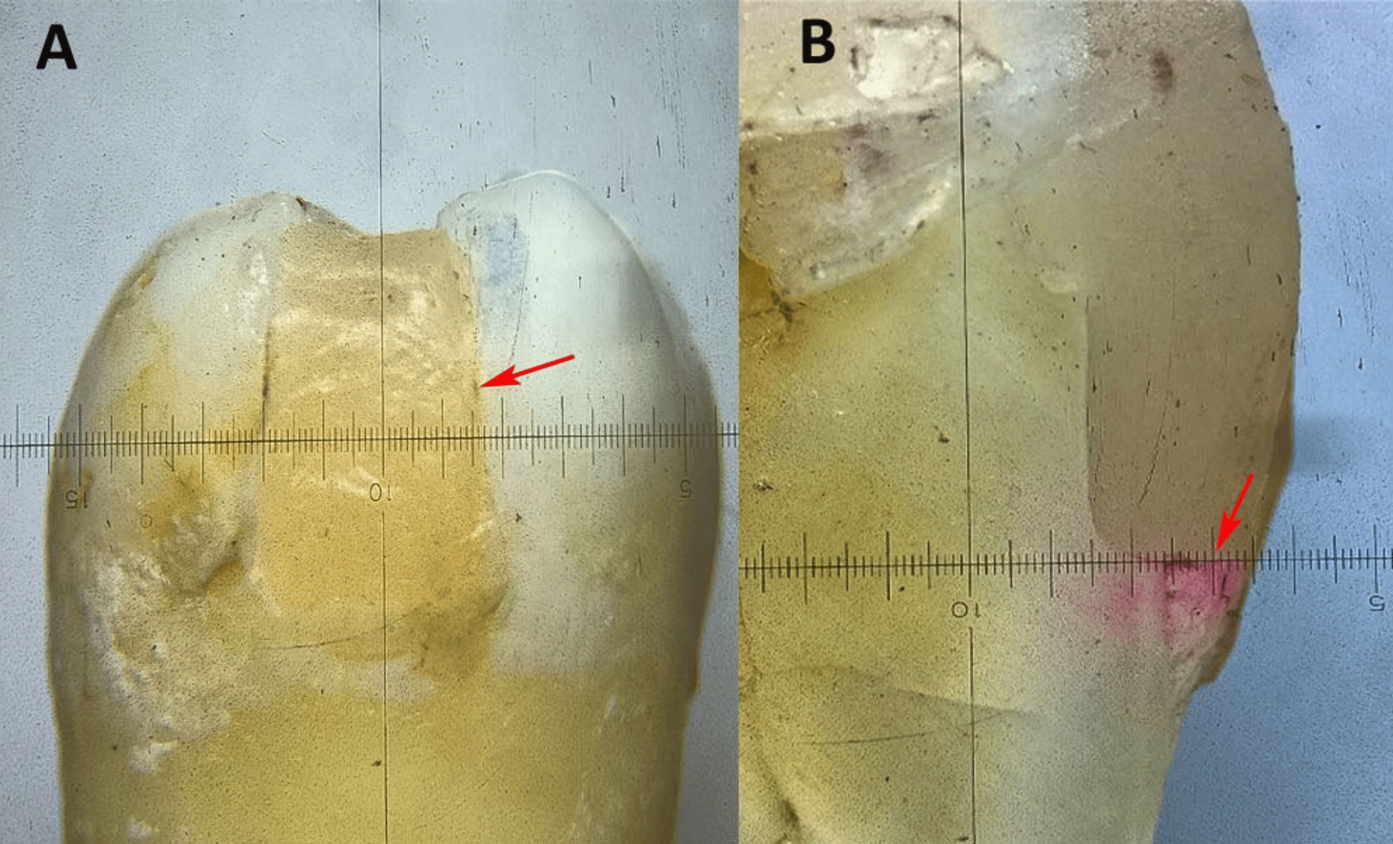
(A) Class II composite filling with gingival floor below the contact area. (B) Mesio-distal section of composite filling with dye penetration at the margin. Figures are at 20x magnification with a stereomicroscope, and the figure depicts data from a sample included in this study.

Upon culmination of the restorations, any excess proximal flash was scrupulously excised using a No. 11 BP scalpel. The apical foramen was occluded with acrylic, and two layers of nail varnish were administered to the dental surface, permitting the restoration and a 2 mm periphery surrounding it to remain exposed. The specimens were subjected to thermocycling (Prevest DenPro Limited, Jammu and Kashmir, India) to replicate oral conditions, alternating between temperatures of 5 ± 1°C and 55 ± 1°C for 1000 cycles, with a dwell duration of 30 seconds at each temperature. After thermocycling, the specimens were preserved in a humidor and submerged in an aqueous 2% erythrosine red dye solution (HiMedia Labs, Maharashtra, India) for 48 hours. Following the immersion procedure, the teeth were meticulously rinsed and bisected in a mesiodistal orientation at the midpoint of the restoration, using a slow-speed, water-cooled diamond disc. The buccal and lingual halves of each tooth section were retained for the analysis (Figure 1A).

The tooth-restoration interface was examined under a stereomicroscope at 20x magnification (RSMr-6 series, Radical Scientific Equipment Pvt. Ltd., Haryana, India) to evaluate the extent of dye penetration (Figure 1B). Observations were scored according to the criteria established by Tredwin et al. [16] to assess microleakage. The scoring system for dye penetration in dental assessments was as follows: a score of 0 indicated no dye penetration; 1 represented dye penetration up to one-third of the gingival seat axially; 2 indicated penetration from one-third to two-thirds of the gingival seat axially; 3 denoted penetration exceeding two-thirds of the gingival seat axially; and 4 signified extensive dye penetration across the entire gingival seat up to the axial wall [16].

Standardization, calibration, and reliability testing

To ensure consistency and accuracy throughout the experimental procedures, several standardization and calibration measures were implemented. All cavity preparations were performed by a single experienced operator to eliminate inter-operator variability. The dimensions of the cavities were verified using a calibrated periodontal probe (UNC-15, Hu-Friedy, Illinois, USA) to maintain uniformity in the cavity size across all samples. The same operator also performed all restorative procedures under magnification (2.5x loupes) to enhance precision. Instrumentation was standardized by replacing the bur after every five cavity preparations, ensuring consistent cutting efficiency and minimizing variations in the cavity surface texture. The light-curing unit (Bluephase N, Ivoclar Vivadent, Schaan, Liechtenstein) was checked for output intensity using a radiometer (Bluephase Meter II, Ivoclar Vivadent) before each curing procedure to ensure consistent light exposure across all the specimens. To calibrate the examiner responsible for the microleakage scoring, a training session was conducted using a separate set of pilot samples (n = 10), which were prepared and evaluated in the same manner as the study samples. To prevent bias, the observer was blinded to the group allocation during the evaluation. For intra-examiner reliability assessment, 15 randomly selected specimens were re-evaluated after a one-week interval. The scores from the two sessions were analyzed using Cohen’s kappa (κ) statistic to determine scoring consistency. A κ value of ≥ 0.80 was considered to represent excellent agreement.

Statistical analysis

The gathered data were meticulously input into Microsoft Excel (Microsoft Corporation, Redmond, WA) for further examination and subsequently analyzed using SPSS version 23.0 software (IBM Corp., Armonk, NY). The distribution of data was evaluated for normality using the Shapiro-Wilk test, and the data were found to be normally distributed. Continuous variables are presented as means and standard deviations (SD). Microleakage scores were subjected to one-way analysis of variance (ANOVA) to determine statistically significant differences among the outcome variables. The confidence interval (CI) for the statistical analyses was 95%. The p-value was set at 0.05.

## Results

The Cohen’s kappa value of 0.88 showed excellent reliability and reproducibility. The results of this study demonstrated significant differences in dye microleakage among the three experimental groups. Group 1 exhibited the highest mean microleakage value (3.35 ± 0.59), indicating greater dye penetration at the tooth-restoration interface. Group 2 showed a moderate reduction in microleakage, with a mean value of 2.30 ± 1.17. In contrast, group 3 had the lowest mean microleakage (1.15 ± 0.93), reflecting superior sealing efficacy. Statistical analysis revealed that the differences in the mean microleakage between the groups were significant (p < 0.05), highlighting the variation in performance among the materials tested (Table 1).

**Table 1:** Comparison of mean scores of microleakage by dye penetration method between different groups by one-way analysis of variance (ANOVA). * P-value < 0.05 is significant. Group 1: control group, restored with Filtek. Group 2: restored with TheraCal LC. Group 3: restored with Bio-LCB. Data are presented as mean and standard deviation (SD).

Groups	n (%)	Mean ± SD	Minimum	Maximum	F stat	p-value
Group 1	20 (33.33%)	3.35 ± 0.58	2	4	28.26	0.001*
Group 2	20 (33.33%)	2.30 ± 1.17	0	4		
Group 3	20 (33.33%)	1.15 ± 0.93	0	3		

The comparative analysis of the mean dye microleakage across the experimental groups indicated statistically significant variations. Group 1 exhibited greater microleakage than group 2 (mean difference: 1.05; p = 0.002) and group 3 (mean difference: 2.20; p = 0.001), signifying deficient sealing capability. Likewise, group 2 demonstrated significantly higher microleakage than group 3 (mean difference: 1.15; p = 0.001). The enhanced effectiveness of groups 2 and 3 compared to group 1 indicated that the utilization of a pulp capping agent as a cavity liner is advantageous in mitigating microleakage (Table 2).

**Table 2:** Pair-wise comparison of groups for microleakage by dye penetration method using post-hoc Tukey's test. * P-value < 0.05 is significant. Group 1: control group, restored with Filtek. Group 2: restored with TheraCal LC. Group 3: restored with Bio-LCB.

Pair-wise comparison		Mean difference	p-value using post-hoc Tukey's test	95% confidence interval	
				Lower bound	Upper bound
Group 1	Group 2	1.050	0.002*	0.34	1.76
Group 1	Group 3	2.200	0.001*	1.49	2.91
Group 2	Group 3	1.150	0.001*	0.44	1.86

## Discussion

Microleakage represents a considerable obstacle in composite restorations, leading to comprehensive investigations over time aimed at alleviating polymerization contraction and consequently diminishing microleakage [2]. Bond failure between the restoration and the tooth margin can lead to significant microleakage. To avoid such problems and relieve the stress caused by polymerization, a highly flexible intermediate layer of liner between the restoration and tooth is recommended [4].

The current investigation was undertaken to evaluate the efficacy of two light-cured pulp capping agents as cavity liners in mitigating microleakage. The primary objective of a pulp capping material is to stimulate distinct hard tissue formation by pulp cells that occlude the site of exposure, thereby promoting continued pulp vitality [6]. Light-cured pulp capping agents have been developed, demonstrating superior marginal adaptation in conjunction with composite restorations [11,13]. These agents incorporated a photoactive moiety into the modified urethane dimethacrylate resin [9]. It has been noted that activated resin exhibits a comparatively slow rate of radical polymerization. Furthermore, the photoinitiators present in the resin played a significant role in the polymerization mechanism. Moreover, the integration of activated resin leads to a reduction of 60-70% in shrinkage stress when juxtaposed with traditional methacrylate-based resins [10].

The current investigation indicated diminished scores for TheraCal LC and Bio-LCB, which can be ascribed to the incorporation of a liner. Liners generally possess a modulus of elasticity that is 20-30% inferior than that of standard hybrid composites [11]. This property helps to mitigate the effects of polymerization shrinkage on cavity walls [9]. Moreover, the application of liners decreased the quantity of composite material utilized in the cavity. This decrease in resin volume leads to a reduction in volumetric shrinkage, consequently minimizing the stress produced and curtailing microleakage [17]. Furthermore, liners obstruct the adhesion of composite resin to dentin. This restriction on the bonded composite surfaces diminished the cavity configuration factor (C-factor). As a result, the tensile stress arising from polymerization shrinkage of the composite is alleviated [18].

Among the groups assessed, TheraCal LC exhibited significantly elevated leakage values compared to Bio-LCB when utilized as a liner beneath class II restorations. This phenomenon may be attributed to the resin matrix contained in the TheraCal LC, which could have altered certain characteristics [11]. Polymerization shrinkage associated with the resin composition may also be a contributing factor [6,7]. TheraCal LC may act as a scaffold for the generation of reparative dentin. The absorption of dentinal fluids within this material facilitates the liberation of calcium and hydroxide ions, prompting the tooth to synthesize apatite and establish a bond, thereby enhancing the inherent sealing capacity of apatite, which is vital for the protection of the pulp [11,19]. TheraCal LC has been documented to possess apatite-forming properties [19]. TheraCal LC is a resin-based material that facilitates the layering of composite resins. The effectiveness of layering TheraCal LC with resin composites, employing either total-etch adhesives or self-etch adhesives in addition to glass ionomers, was investigated by Meraji and Camilleri [20].

Bio-LCB, as a light-cure pulp capping agent, showed minimal microleakage compared to TheraCal LC. This is because TheraCal LC contains bisphenol A-glycidyl methacrylate (BIS-GMA), while Bio-LCB contains urethane dimethacrylate (UDMA), which has a lower probability of polymerization shrinkage [13]. Compared to other materials, UDMA-based materials often have lower shrinkage rates upon polymerization. This reduced shrinkage can help minimize gaps between the filling and tooth, thereby reducing the likelihood of microleakage [21]. UDMA-based materials can offer high mechanical strength and durability to dental restorations that experience chewing and biting forces. A strong restoration is less likely to fracture or degrade over time, reducing the chances of gap formation where microleakage can occur [21]. UDMA-based materials often have good adhesive properties, allowing them to form strong bonds with tooth structures. A reliable bond helps to create a tight seal at the restoration-tooth interface, minimizing opportunities for microleakage [22]. This elucidates the rationale for the diminished leakage values observed beneath the Bio-LCB in class II composite restorations within this investigation.

Bio-LCB bioactive constituents establish more robust adhesion to dentin. This chemical affinity to the dental substrate surpasses that of non-bioactive substances such as TheraCal LC, resulting in a more resilient seal and diminishing conduits for fluid or bacterial penetration into the restoration [23]. Bio-LCB experiences minor expansion during the curing process, facilitating enhanced conformance to the cavity walls. This phenomenon reduces the probability of void formation between the restorative material and tooth surface, thereby mitigating the risk of microleakage [24].

Clinical implications

The clinical implications of this research underscore the significance of choosing bioactive materials such as Bio-LCB to mitigate microleakage and improve the durability of restorations. These results indicate that Bio-LCB possesses enhanced sealing capabilities relative to materials such as TheraCal LC, potentially resulting in superior defense against secondary caries and increased restoration efficacy. These findings highlight the prospective advantages of employing bioactive liners in endodontic and restorative interventions, particularly in molar and premolar dentition, to prevent microleakage and foster long-term success. This investigation advocates the continued exploration of bioactive materials aimed at refining restorative treatment methodologies in clinical environments.

Limitations

The constraints of this investigation include the employment of a single tooth type (mandibular premolars), which may inadequately reflect the heterogeneity of the dental morphology encountered in clinical scenarios. The in vitro design of the study constrains the relevance of the findings to actual clinical situations, as the exclusion of biological factors such as pulp vitality and the oral environment could potentially influence the results. Moreover, the brief duration of thermocycling may not fully replicate long-term clinical conditions. Although the dye penetration technique is efficacious, it is inherently subjective and may not accurately depict the true microleakage dynamics in a clinical context.

## Conclusions

The findings of the current investigation demonstrated that the application of light-cured pulp capping materials as cavity liners significantly diminished microleakage. The measurements of microleakage were particularly favorable with Bio-LCB as a lining agent compared with TheraCal LC. It can be inferred that both Bio-LCB and TheraCal LC may be regarded as effective candidates for lining extensive class II cavities that require pulp capping interventions. However, it is important to note that none of the evaluated materials or methodologies completely eliminated microleakage at the interfaces of class II composite restorations.
